# Differential regulation of miRNA and mRNA expression in the myocardium of Nrf2 knockout mice

**DOI:** 10.1186/s12864-017-3875-3

**Published:** 2017-07-03

**Authors:** Justin M. Quiles, Madhusudhanan Narasimhan, Gobinath Shanmugam, Brett Milash, John R. Hoidal, Namakkal S. Rajasekaran

**Affiliations:** 1 Cardiac Aging & Redox Signaling Laboratory, Division of Molecular & Cellular Pathology, Department of Pathology, University of Alabama at Birmingham, BMR2 Room 533|901 19th Street South, Birmingham, AL 35294-2180 USA; 2 Department of Pharmacology and Neuroscience, Texas Tech University Health Sciences Center, Lubbock, TX 79430 USA; 3 Huntsman Cancer Institute, Salt Lake City, USA; 4 Division of Pulmonary, Salt Lake City, USA; 5 Cardiovascular Medicine, Department of Medicine, University of Utah School of Medicine, Salt Lake City, UT 84132 USA; 6 Center for Free Radical Biology, University of Alabama at Birmingham, BMR2 Room 533|901 19th Street South, Birmingham, AL 35294-2180 USA

**Keywords:** Nrf2, microRNA, mRNA, Heart, RNA sequencing, Differential expression, Antioxidant, Redox

## Abstract

**Background:**

Nuclear Factor Erythroid-derived 2-like 2 (Nrf2) senses oxidative environments and/or stress and initiates a cytoprotective response through transcriptional activation of antioxidant and detoxification genes. Several preclinical studies suggest that Nrf2 combats oxidative stress underlying a variety of pathologies. Despite Nrf2 deficits linked to functional abnormalities in many organ systems, the transcriptional network resulting from Nrf2 deficiency in the heart has remained elusive. Moreover, cross-talk between microRNAs (miRNAs) and cardiac Nrf2 signaling is unknown. Here, we utilized next generation RNA sequencing (RNAseq) to unbiasedly profile basal mRNA and miRNA expression in Nrf2 knockout (Nrf2^−/−^) hearts.

**Results:**

RNAseq of mRNA revealed 152 differentially expressed genes (DEGs) in the Nrf2^−/−^myocardium, of which 129 were downregulated. Grouping of DEGs based on biological function and real-time qPCR validation indicated that DEGs were enriched for; mitochondrial genome and bioenergetics, oxidoreductase capacity, cardiac development, and chaperone activity. Interestingly, RNAseq analysis uncovered 27 significantly altered miRNAs, of which 11 were upregulated and 16 were downregulated in Nrf2^−/−^ hearts. Expression changes were validated for 12 miRNAs using specific primer assays in real-time and revealed a significant decrease in miR-10b-5p, miR-674-3p, miR-3535, and miR-378c while miR-30b-5p, miR-208a-5p, miR-350-3p, and miR-582-5p, and miR-1249-3p levels were increased. High throughput data were integrated using prediction algorithms, and these in silico analyses discovered potential recognition elements within 39 repressed mRNAs which matched the seed sequence for 4 upregulated miRNAs; miR-30b-5p, miR-208a-5p, miR-350-3p, and miR-582-5p.

**Conclusion:**

These high-throughput data reveal transcriptome-wide effects of myocardial Nrf2 deficiency. Further, our results suggest that Nrf2 may directly or indirectly regulate a sub-set of cardiac miRNAs in the basal setting. This comprehensive analysis is the first evidence to demonstrate a plausible regulatory cross-talk among cardiac miRNAs and the Nrf2 transcriptional network, and provides valuable candidates to examine in future mechanistic and preclinical studies.

**Electronic supplementary material:**

The online version of this article (doi:10.1186/s12864-017-3875-3) contains supplementary material, which is available to authorized users.

## Background

Nuclear factor erythroid 2 like 2 (NFE2L2/Nrf2) is a conserved cap‘n’collar (CNC) basic leucine zipper transcription factor which affords cytoprotection against xenobiotics and reactive oxygen species (ROS) through induction of antioxidant (ARE) and electrophile (EpRE) response elements [1]. Since its discovery, nearly 2 decades of research has delineated beneficial roles for Nrf2 mediated transcriptional programs in various oxidative stress-related disease models, such as chronic obstructive pulmonary disease (COPD), neurodegeneration, inflammation, carcinogenesis, and pathogenesis associated with environmental toxicant exposure [2–6]. Along these lines, numerous investigations have clearly demonstrated pathological susceptibility of the genetically Nrf2 ablated (Nrf2^−/−^) mouse to exogenous oxidative challenges [1]. However, apart from our laboratory [7, 8], few investigations have explored the role of Nrf2 in the heart [9, 10]. Importantly, the myocardium is exquisitely sensitive to redox disturbances, and oxidative stress has been directly linked to the pathophysiological mechanisms of heart failure [11]. While basal myocardial antioxidant expression and ROS are comparable between young Nrf2^−/−^ and age matched wild-type (WT) counterparts, knockout hearts are unable to cope with the oxidative burden induced by high intensity exercise and aged Nrf2^−/−^ mice exposed to such stress exhibit pathological atrial hypertrophy [7, 8].

Although Nrf2 is ubiquitously expressed, the cytoprotective signaling cascade is believed to be quiescent without the appropriate redox-dependent catalyst [1]. Basal repression of Nrf2 nuclear import is facilitated by Kelch like ECH associated protein 1 (Keap1) [12]. The Keap1 homodimer anchors Nrf2 to the actin cytoskeleton serving as a substrate adaptor for the Cul3 ligase which promotes rapid ubiquitination of Nrf2, and subsequent degradation by the 26S proteasome [13, 14]. However, upon redox perturbation, a number of crucial cysteine thiols in Keap1 are directly modified by electrophiles to permit Nrf2 dissociation and nuclear translocation [15]. In addition to the classic model of Keap1 mediated repression, recent studies have discovered translational regulation of Nrf2 within its open reading frame [16], as well as non-coding RNA regulation at the 3′ untranslated region [17]. Collectively, these mechanisms work in concert to account for low levels of functional Nrf2 protein at baseline.

MicroRNAs (miRNAs) are potent regulators of gene expression that govern numerous aspects of cellular function through their recognition of complementary sequences in target mRNAs [18]. Despite consistent reports of aberrant miRNA expression patterns in disease progression [19, 20], few studies have explored cross-talk between Nrf2 signaling and miRNA transcriptional responses. Further, existing data largely centers on cancer pathogenesis [21]. The increasing utility of miRNA signatures in the prediction and prognosis of cardiac diseases [22, 23], coupled with relevance of oxidative stress in these pathologies has created a need for investigating the myocardial miRNA transcriptome amidst Nrf2 deletion.

Accordingly, we have performed high throughput RNA sequencing (RNAseq) using the hearts of WT and Nrf2^−/−^ mice to explore mRNA and miRNA expression patterns and elucidate regulatory networks arising from loss of Nrf2 in the myocardium. Knockout mice showed modest downregulation in a number of redox and cardiac pathology transcripts previously unidentified. For the first time, we have uncovered a distinct miRNA signature in the Nrf2^−/−^ myocardium. Strikingly, 27 miRNAs (11 up and 16 downregulated) were found to be significantly altered at baseline in the heart when Nrf2 is genetically disrupted. In silico target prediction for increased miRNAs suggested that many mRNAs altered in knockout mice may be concurrently regulated by miR-582-5p, miR-208a-5p, miR-350-3p, and miR-30b-5p. Considering the pathological susceptibility of Nrf2^−/−^ hearts, we believe these miRNAs could be valuable predictive indicators of myocardial oxidative stress commonly associated with cardiac pathophysiology.

## Methods

### Animals

Adult C57BL/6 J (WT) and Nrf2 knockout (Nrf2^−/−^) mice 6–8 months of age were used for gene expression studies to examine transcriptional consequences of Nrf2 deletion in the heart, and observe the role of microRNAs (miRNAs) in myocardial oxidative stress. The knockout mice were genotyped as we previously described [7]. Animals were housed pathogen free and maintained in a temperature controlled environment with a 12 h day/night cycle and given access to standard rodent chow and water ad libitum, and were humanely treated in accordance with the Guide for Care and Use of Laboratory Animals developed by the National Research Council at the National Institutes of Health (NIH). All experiments were approved by the Institutional Animal Care and Use Committees (IACUC) at the University of Utah and the University of Alabama at Birmingham.

### Next generation mRNA sequencing

Cardiac RNA was isolated from 6 to 8 month male WT and Nrf2^−/−^ (*n* = 3–4/group) using the RNeasy Mini Kit (Qiagen, Cat. 74,106) according to the manufacturer’s instructions. After confirmation of sample purity, intact poly(A) transcripts were purified from total RNA using oligo(dT) magnetic beads and mRNA sequencing libraries were prepared with the TruSeq Stranded mRNA Library Preparation Kit (Illumina, RS-122-2101, RS-122-2102). A D1000 ScreenTape assay (Agilent, Cat. 5067–5582/3) was used with the 2200 TapeStation Instrument (Agilent Technologies) to qualify purified libraries. The cBot was used to apply 18pM of the sequencing library to a TruSeq v3 flowcell (Illumina) and the TruSeq SR Cluster Kit (Illumina, Cat. GD-401-3001) was used for clonal amplification. Finally, the flowcell was transferred to the HiSeq 2000 instrument and used in 50 cycle single read sequence run performed with TruSeq SBS Kit v3-HS reagents (Illumina, Cat. FC-401-3002). Novoindex (2.8) was used to create a reference index on a combination of hg19 chromosome and splice junction sequences. Splice junction sequences were generated with USeq (v8.6.4) MakeTranscriptome using Ensembl transcript annotations (build 67). Reads were aligned to the transcriptome reference index described above using Novoalign (v2.08.01), allowing up to 50 alignments for each read. USeq’s SamTranscriptomeParser application was used to select the best alignment for each read and convert the coordinates of reads aligning to splices back to genomic space. Differential gene expression was measured using USeq’s DefinedRegionDifferentialSeq application. The number of reads aligned to each gene was calculated. The counts were then used in DESeq (v1.24.0), which normalizes the signal and determines differential expression [24].

### Next generation miRNA sequencing

Mature miRNA transcripts were extracted from 6 to 8 month WT and Nrf2^−/−^ (*n* = 3/group) hearts using the miRNeasy Kit (Qiagen, Cat. 217,004). After confirmation of sample purity, the NEBNext Multiplex Small RNA Library Prep Set for Illumina (NEB, Cat E7300) was used to prepare small RNA sequencing libraries and adapter-ligated molecules encoding small RNAs were enriched. Pippin Prep size selection (Sage Science) with 3% agarose was performed according to the following paramters: BP Start = 105 bp, BP End = 155 bp. The cBot was used to apply 25pM of the sequencing library to a HiSeq v4 flowcell (Illumina) and the HiSeq SR Cluster Kit (Illumina, Cat. GD-401-4001) was used for clonal amplification. Finally, the flowcell was transferred to the HiSeq 2500 instrument and used in 50 cycle single read sequence run performed with HiSeq SBS Kit v4 reagents (Illumina, Cat. FC-401-4002). Mouse Ensembl gene annotations (build 74) were downloaded and converted to genePred format. Splice junction sequences were generated using USeq’s (v8.8.9) MakeTranscriptome application using a radius of 46. These splice junction sequences were added to the mouse chromosome sequences (mm10) and run through novoindex (v2.8) to create the transcriptome index. Reads were aligned to the transcriptome reference index with Novoalign (v2.08.03), allowing up to 50 alignments for each read. USeq’s SamTranscriptomeParser application was used to select the best alignment for each read and convert the coordinates of reads aligning to splices back to genomic space. Read counts for each mature miRNA (mirBase v21) were generated using USeq’s DefinedRegionDifferentialSeq application. These counts were used in DESeq2 to measure the differential expression between each group.

### RNA isolation, reverse transcription and real-time quantitative PCR

Approximately 10-25 mg of snap-frozen heart tissue (*n* = 4–6/group) were homogenized (Bio-Gen PRO200, PRO Scientific Inc) in QIAzol lysis reagent (Qiagen, Cat. 79,306) and RNA was extracted using the miRNeasy Mini Kit (Qiagen, Cat. 217,004). Following isolation, RNA was quantified and assessed for purity with the NanoDrop One^C^ Spectrophotometer (ThermoFisher Scientific). 1-2 μg RNA was reverse transcribed to synthesize cDNA using either the QuantiTect Kit (Qiagen, Cat. 205,313) or miScript II RT Kit (Qiagen, 218,161) for mRNA and miRNA qPCR, respectively. HiSpec buffer was used in cDNA synthesis for downstream mature miRNA quantification. In both applications, real-time qPCR analysis was carried out in a 10 μl reaction volume containing cDNA template, respective primer sets (Tables 1 and 2) and QuantiTect SYBR Green master mix (Qiagen) in a Roche LightCycler 480 (Roche Life Science). Target gene mRNA levels were quantified using Ct values, and relative expression (fold change) was calculated by normalization to the Ct of housekeeping genes according to the 2^-ΔΔCt^ method. *Rplp0* (*Arbp1*) and *Gapdh* were used as housekeeping genes for mRNA studies while the small nucleolar RNA, C/D box 42B (*Snord42B*) was used to quantify miRNA expression.

**Table 1:** mRNA RT-qPCR Primers: Complete list of all mRNA primers used in real-time qPCR experiments

Complete List of mRNA Primer Sequences		
Gene Symbol	Forward	Reverse
Nrf2	CTGAACTCCTGGACGGGACTA	CGGTGGGTCTCCGTAAATGG
Mgstl	GACAACTTGCAGCCCTTCTC	ATTGTCCATGAGCTGCCTGA
Srxnl	CCCAGGGTGGCGACTACTA	GTGGACCTCACGAGCTTGG
Rcanl	GACACAGTGCCTTTCCCCTT	CACACACACGATGACTGGGA
Abra	ATCGAGACGGAGAGGGACAA	TTGCTGACAACCGTTCTGGT
Atf3	AGAGTCTGGGGATCTGCCAT	GTTGGCACAAAGTGGCTCAG
Hsphl	TAAGGCTGAGCGATTGGGAC	GAAGATGCCTCCGGCTTACC
Adamtsl	AGCCCAAGGTTGTAGATGGC	CACAGCCAGCTTTCACACAC
Nnt	GCAGAGACAAAGGGGCTTCA	AAGACTCCACCAGATGCACG
mt-Nd4l	ACACTTCTATGACAAACCGACGA	TGTGGATAGGGGGTCTGAGG
mt-Nd6	AGGACTGGAATGCTGGTTGG	ACCCAATCAAACGCCTAGCA
Gstal	AGCCCGTGCTTCACTACTTC	TCTTCAAACTCCACCCCTGC
Osginl	GGGCTAATGGGCATCCTGTT	CAGCTGACCTGATGGATGGT
Aoxl	CAGAACGGAAGCTGGAGTGT	CCTGGCCGCCTATGTGTATT
Gck	AGGAGGCCAGTGTAAAGATGT	CTCCCAGGTCTAAGGAGAGAAA
Ctgf	AGACCTGTGCCTGCCATTAC	ACGCCATGTCTCCGTACATC
Acta2	CCCTGGAGAAGAGCTACGAAC	TTTCGTGGATGCCCGCTG
Ankrdl	TGCGATGAGTATAAACGGACG	GTGGATTCAAGCATATCTCGGAA
Thbsl	GTGAGGTTTGTCTTTGGAACCA	GTTGTTGTCAAGGGTAAGAAGGA
Stipl	CCCTGAGTGCTGGGAACATT	TCCI1ICIIGGCGTAGGCTG
Dnajb4	GCGTGGCGACCTACTGATAG	TAGGAGGCAGGGAGATGCTT
Gstml	GATTGGTGCAGGGTTGGGAG	GCTGGTGCTGTGGTCTTCTC
RplpO	TGAGATTCGGGATATGCTGTTGG	CGGGTCCTAGACCAGTGTTCT
Gapdh	TGACCTCAACTACATGGTCTACA	CTTCCCATTCTCGGCCTTG

**Table 2:** miRNA RT-qPCR Primers: Catalog numbers for all miRNA miScript primer assays (Qiagen)

miScript miRNA Primer Assays	
Gene Symbol	Catalog #
mmu-miR-27a-5p	MS00011564
mmu-miR-10b-5p	MS00032249
mmu-miR-674-3p	MS00012495
mmu-miR-3535	MS00065463
mmu-miR-1964-3p	MS00024444
mmu-miR-378c	MS00064623
mmu-miR-582-5p	MS00012383
mmu-miR-1983	MS00016884
mmu-miR-208a-5p	MS00024542
mmu-miR-350-3p	MS00007938
mmu-miR-1249-3p	MS00024045
mmu-miR-30b-5p	MS00001386
Snord42B	MS00055090

### Bioinformatic prediction of miRNA targets and STRING analysis

Since computational algorithms place varying degrees of emphasis on seed match, conservation, free energy, and site accessibility in predicting miRNA recognition elements [25], we employed 6 different programs to uncover potential miRNA:mRNA interactions in Nrf2^−/−^ hearts. These included; miRANDA-mirSVR, TargetScan, MirTarget2 (miRDB), DIANA-microT-CDS, rna22, and Probability of Interaction by Target Accessibility (PITA). When using miRANDA-mirSVR, mRNAs were matched only to miRNAs with ‘good’ SVR scores (≤ − 0.1). For TargetScan analysis, version 7.1 was used with the murine genome. When browsing the mouse DIANA-microT-CDS interface, threshold was set to 0.7. Finally, minimum seed size for the PITA tool was set to 7 and permitted to allow a single mismatch and G:U wobble. This systemic approach was taken for downregulated differentially expressed genes (DEGs) revealed through RNA sequencing in an effort to associate repressed mRNAs with basally upregulated miRNAs in the Nrf2^−/−^ myocardium. All mRNAs potentially targeted by miRNAs (see Fig. 5) were ran in the Search Tool for the Retrieval of Interacting Genes/Proteins (STRING) database to create a gene network based on the following active interaction sources; text mining, neighborhood, experimental evidence, additional database support, co-expression studies, gene-fusion and co-occurrence. Minimum required interaction score was set to 0.400 and the network was drawn whereby line thickness indicates strength of data to support the interaction. Disconnected nodes were omitted from the network display. Functional enrichment analysis was done for the network obtained from STRING to reveal biological processes implicated in miRNA:mRNA interactions. Gene Ontology (GO) designations for biological processes were plotted according to the log2 transformed false discovery rates (FDR).

### Statistical analyses

Gene expression fold change was calculated according to the 2^-ΔΔCt^ method and group (*n* = 4–6) data are presented as mean ± SEM. Basal comparisons between wild-type and knockout mice were made using an unpaired t-test. All statistical analyses were performed using GraphPad Prism software with significance set to *p* < 0.05.

## Results

### Cardiac Transcriptome in Nrf2 knockout mice

**Fig. 1:**
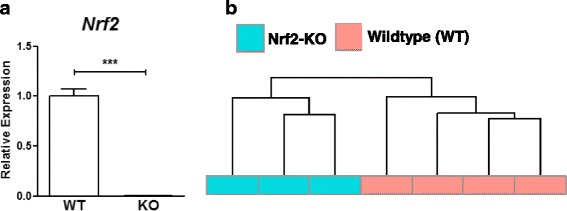
Nrf2 Deletion and Sample Clustering: **a** Real-time quantitative PCR with cardiac RNA confirms Nrf2 ablation in knockout hearts (*n* = 4–6/group). **b** Hierarchal clustering analysis for all samples used in next-generation RNA sequencing (RNAseq) (*n* = 3–4/group). Clustering was performed for all genes with FPKM ≥1 in at least two samples and revealed distinct grouping of wild-type and knockout genotypes. ***p < 0.001

**Fig. 2:**
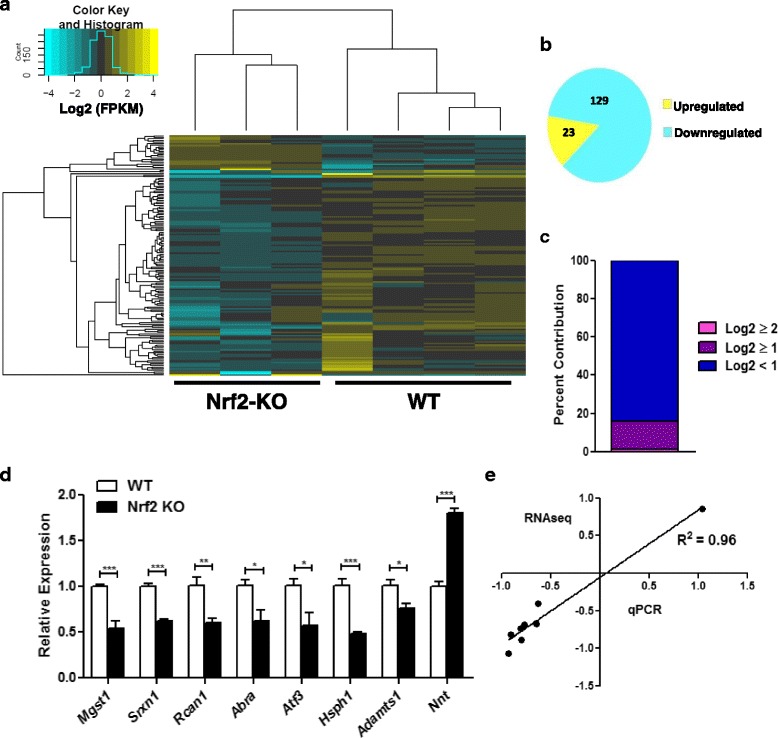
Cardiac Transcriptome in Nrf2 Knockout Mice: **a** Hierarchal clustering of 152 differentially expressed genes (DEGs) in Nrf2 KO hearts uncovered by RNA sequencing (RNAseq) (*n* = 3–4/group). **b** 129 DEGs were downregulated while only 23 were upregulated. **c** DEGs separated by log2 fold change (FC) showed that most DEGs yielded a log2 FC less than 1.0 (84%). Apart from Nrf2 itself, just 22 (15%) and 2 (1%) DEGs revealed a log2 FC greater than 1.0 and 2.0, respectively. **d-e** Real-time quantitative PCR (*n* = 4–6/group) of 8 DEGs shows a strong correlation with RNAseq log2FC. *p < 0.05, **p < 0.01, ***p < 0.001

High throughput sequencing was performed using cardiac RNA from Nrf2^−/−^ mice (Fig. 1a). Importantly, hierarchal clustering for all genes indicated a distinct grouping of the knockout and control genotypes (Fig. 1b). Sequencing analysis revealed a total of 152 differentially expressed genes (DEGs) in the hearts of knockout mice (Fig. 2a, Additional file 1: Table S1) in which 129 transcripts showed decreased abundance, while only 23 genes exhibited increased expression (Fig. 2b). Overall, the majority of DEGs discovered in the Nrf2 ablated myocardium displayed only modest expression changes as 84% of all DEGs were assigned a log2 fold change (FC) less than 1.0. Furthermore, 22 DEGs demonstrated a log2 FC greater than 1.0 and apart from Nrf2 itself, only 2 DEGs (*Gm12913* and *Xist*) revealed a log2 FC greater than 2.0 (Fig. 2c). Importantly, real-time quantitative polymerase chain reaction (qPCR) for the 8 genes selected showed exceptional consistency with our RNA sequencing analysis (Fig. 2d and e).

### Mitochondrial, Redox and metabolic transcriptional signature

**Fig. 3:**
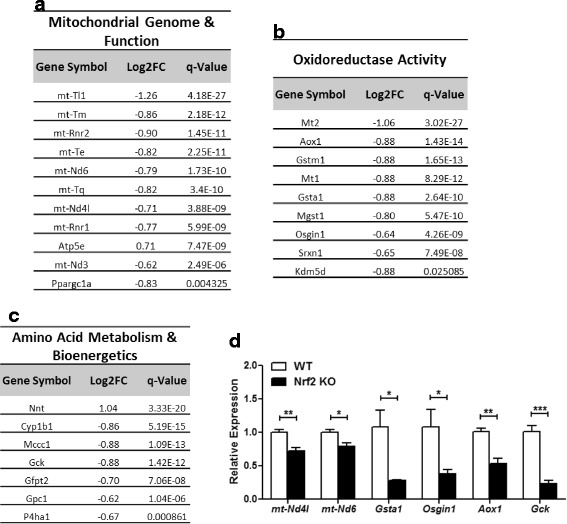
Mitochondrial, Redox and Metabolic Transcriptional Signature: DEGs obtained from cardiac RNA sequencing (RNAseq) partitioned into the following biological categories using Gene Ontology and Reactome pathway designations; (**a**) Mitochondrial Genome and Function, (**b**) Oxidoreductase Activity, and (**c**) Amino Acid Metabolism and Bioenergetics. Log2 fold change (Log2FC) derived by RNAseq is provided for each gene along with an adjusted *p*-value to account for false discovery rate (q-value). **d** Real-time quantitative PCR validation (*n* = 4–6/group) of 6 genes representing each major biological pathway. *p < 0.05, **p < 0.01, ***p < 0.001

After analyzing and validating RNA sequencing data from Nrf2^−/−^ mouse hearts we examined the pool of DEGs and assigned genes to their overarching biological category using Gene Ontology and Reactome pathway annotations [26, 27]. Fig. 3a-c rank gene lists according to the false discovery rate adjusted *p*-value (q-value) derived from sequencing, and provide corresponding expression changes for each transcript. A substantial number of genes encoded within the mitochondrial genome were decreased in knockout mice including mitochondrial tRNAs leucine 1 (*mt-Tl1*), methionine (*mt-Tm*), glutamic acid (*mt-Te*), and glutamine (*mt-Tq*), as well as 12S and 16S rRNAs (*mt-Rnr1*, *mt-Rnr2*). Further, NADH dehydrogenase subunits 3, 4 L and 6 (*mt-Nd3*, *mt-Nd4l*, *mt-Nd6*) were significantly repressed in the Nrf2^−/−^ myocardium (Fig. 3a). Real-time qPCR confirmed *mt-Nd4l* and *mt-Nd6* expression and they were significantly decreased by 28% (*p* < 0.01) and 21% (*p* < 0.05) in knockout mice, respectively (Fig. 3d). The decrease in peroxisome proliferative activated receptor, gamma, coactivator 1 alpha (*Ppargc1a/PGC-1*α) may explain the marked reduction in these mitochondrially encoded transcripts as *Ppargc1a* is a master transcriptional co-activator of cardiac mitochondrial biogenesis [28] (Fig. 3a).

Indeed, the comprehensive sequencing data presented here depict a minor role for Nrf2 in regulating basal antioxidant expression in the unstressed myocardium as none of the aforementioned transcripts were found to be significantly altered. Moreover, relatively few endogenous antioxidant genes were repressed following sequencing analysis; these included glutathione S-transferases mu 1, alpha 1 and microsomal glutathione S-transferase 1 (*Gstm1*, *Gsta1, Mgst1*) as well as sulfiredoxin 1 (*Srxn1*) (Fig. 3b). Real-time quantification of *Gsta1* mRNA revealed a robust 3.5-fold (*p* < 0.05) decrease (Fig. 3d). While some antioxidant transcripts were repressed upon Nrf2 ablation, the typically expected increase in genes involved in oxidative mechanisms were not apparent. This was evident from the decreased expression of oxidative stress induced growth inhibitor 1 (*Osgin1/OKL38*), a gene that increases cellular levels of ROS [29] and aldehyde oxidase 1 (*Aox1*) (Fig. 3b), a molybdoenzyme family member that can enhance superoxide formation by hydroxylating heterocycles and oxidizing aldehydes into carboxylic acids [30]. Therefore, the 2.2 and 2.6-fold decrease in *Aox1* and *Osgin1*, respectively (Fig. 3d), coupled with an unchanged ROS scavenging transcriptome suggests the absence of oxidative stress in the Nrf2 ablated myocardium under basal setting.

In addition to mitochondrial genome and oxidoreductase genes, RNA sequencing of the Nrf2^−/−^ myocardium revealed DEGs enriched for cellular bioenergetics and amino acid metabolism (Fig. 3c). Surprisingly, the expression of the glycolytic enzyme glucokinase (*Gck*) and glutamine fructose-6-phosphate transaminase 2 (*Gfpt2/GFAT*), a gene involved in glucose sensing via hexosamine biosynthetic pathway, was significantly reduced in Nrf2 knockout hearts relative to wild-type controls (Fig. 3c and d). At present, the significance of cardiac *Gck* expression is not clear. While loss of *Gck* in hepatocytes and β cells is linked to hyperglycemia [31], altered *Gfpt2* expression in Nrf2^−/−^ mice may counterbalance this effect and maintain cardiac glucose homeostasis [32]. The xenobiotic metabolizing enzyme cytochrome P450 family 1 subfamily B member 1 (*Cyp1b1*) was also downregulated in Nrf2^−/−^ mice (Fig. 3c). Interestingly, RNAseq followed by qPCR validation revealed that nicotinamide nucleotide transhydrogenase (*Nnt*), a mitochondrial membrane protein important for combating oxidative burden [33], was significantly increased by 80% (*p* < 0.001) (Figs. 3c and 2d). Collectively, these results suggest that disruption of Nrf2 does not significantly alter basal antioxidant transcript abundance in the myocardium. However, bioenergetic and mitochondrial alterations upon Nrf2 deletion under stress conditions could be detrimental considering the energy demands of the myocardium which warrants further investigation.

### Cardiac markers of stress and Pathophysiology

**Fig. 4:**
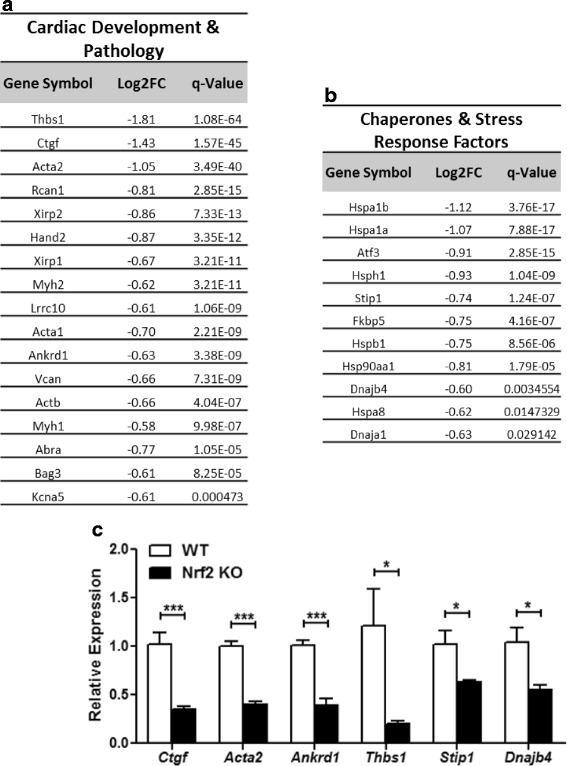
Cardiac Markers of Stress and Pathophysiology: DEGs obtained from cardiac RNA sequencing (RNAseq) separated according biological categories using Gene Ontology and Reactome pathway designations; (**a**) Cardiac Development and Pathology, and (**b**) Chaperones and Stress Response Factors. Log2 fold change (Log2FC) derived by RNAseq is provided for each gene along with an adjusted *p*-value to account for false discovery rate (q-value). (**c**) Real-time quantitative PCR validation (*n* = 4–6/group) of 6 genes representing each major biological pathway. *p < 0.05, **p < 0.01, ***p < 0.001

Figure 4 displays the continuation of DEG assignment to biological categories using Gene Ontology and Reactome pathway annotations. Here, we observed DEGs distinctly enriched for cardiac development and pathology pathways (Fig. 4a), as well as molecular chaperones and stress response signaling (Fig. 4b). Ankyrin repeat domain 1 (*Ankrd1*), a transcriptional regulator of Gata4 signaling which is consistently increased in human heart failure [34, 35], was found to be diminished by 2.5-fold (*p* < 0.001) in knockout heart tissue (Fig. 4c). Several sarcomeric genes, including, xin actin-binding repeat containing 1 (*Xirp1*), actin, alpha 1, skeletal muscle (*Acta1*), actin beta (*Actb*), actin-binding Rho activating protein (*Abra*), as well as myosin heavy chains 1 and 2 (*Myh1*, *Myh2*) were found to be downregulated in Nrf2^−/−^ hearts. In addition, knockout hearts displayed reduced expression of heart and neural crest derivatives expressed transcript 2 (*Hand2*), another transcriptional activator of cardiac development [36] (Fig. 4a). In contrast to these dampened cardiogenic expression programs, the inhibitor of pro-hypertrophic NFAT signaling [37], regulator of calcineurin 1 (*Rcan1*/*DSCR1*/*MCIP1*) was decreased in Nrf2^−/−^ hearts (Figs. 2d and 4a). Importantly, this may represent impaired regulation of calcineurin-dependent circadian rhythmicity, and could likely serve as an index for cardiac vulnerability to reperfusion injury [38].

The Nrf2^−/−^ myocardium displayed a striking 4.9-fold decrease (*p* < 0.05) in thrombospondin 1 (*Thbs1*) expression (Fig. 4c). Despite a lack of obvious basal phenotype, the Thbs1^−/−^ mouse is sensitive to myocardial infarction [39]. This likely indicates that the Nrf2^−/−^ mouse may be susceptible to increased cardiac pathology. Further, the Nrf2^−/−^ myocardium exhibited a 2.8 and 2.5-fold (*p* < 0.001) decrease in the mRNA expression of connective tissue growth factor (*Ctgf*) and actin, alpha 2, smooth muscle, aorta (*Acta2*) (Fig. 4c), two genes increased with TGF-β signaling in cardiac fibrosis [40–42]. This suggests that abrogation of Nrf2 did not evoke any pro-fibrotic signaling events in the heart at baseline.

Recent evidence suggests that redox state and proteostasis are intimately interconnected [43]. Here, Nrf2 deletion resulted in widespread downregulation of several heat shock family member genes including heat shock proteins 1, 1A, 1B, 8 (*Hspb1*, *Hspa1a*, *Hspa1b*, *Hspa8*), heat shock protein 90, alpha, (cytosolic), class A member 1 (*Hsp90aa1*), heat shock 105 kDa/110 kDa protein 1 (*Hsph1*), as well as DnaJ heat shock protein family (Hsp40) members A1 and B4 (*Dnaja1, Dnajb4*). Furthermore, co-chaperones stress-induced phosphoprotein 1 (*Stip1*) and FK506 binding protein 5 (*Fkbp5*) were also reduced in knockout hearts (Fig. 4b). This expression pattern observed through RNA sequencing was validated with primer assays specific for *Stip1* and *Dnajb4*, and indeed mRNA levels decreased significantly by 1.6 and 1.8-fold (*p* < 0.05), respectively (Fig. 4c). The general decrease in heat shock family genes confirms the role of Nrf2 as a highly conserved unfolded protein response factor whose transcriptional program overlaps with Hsf1 [44].

### Myocardial microRNA expression profile

**Fig. 5:**
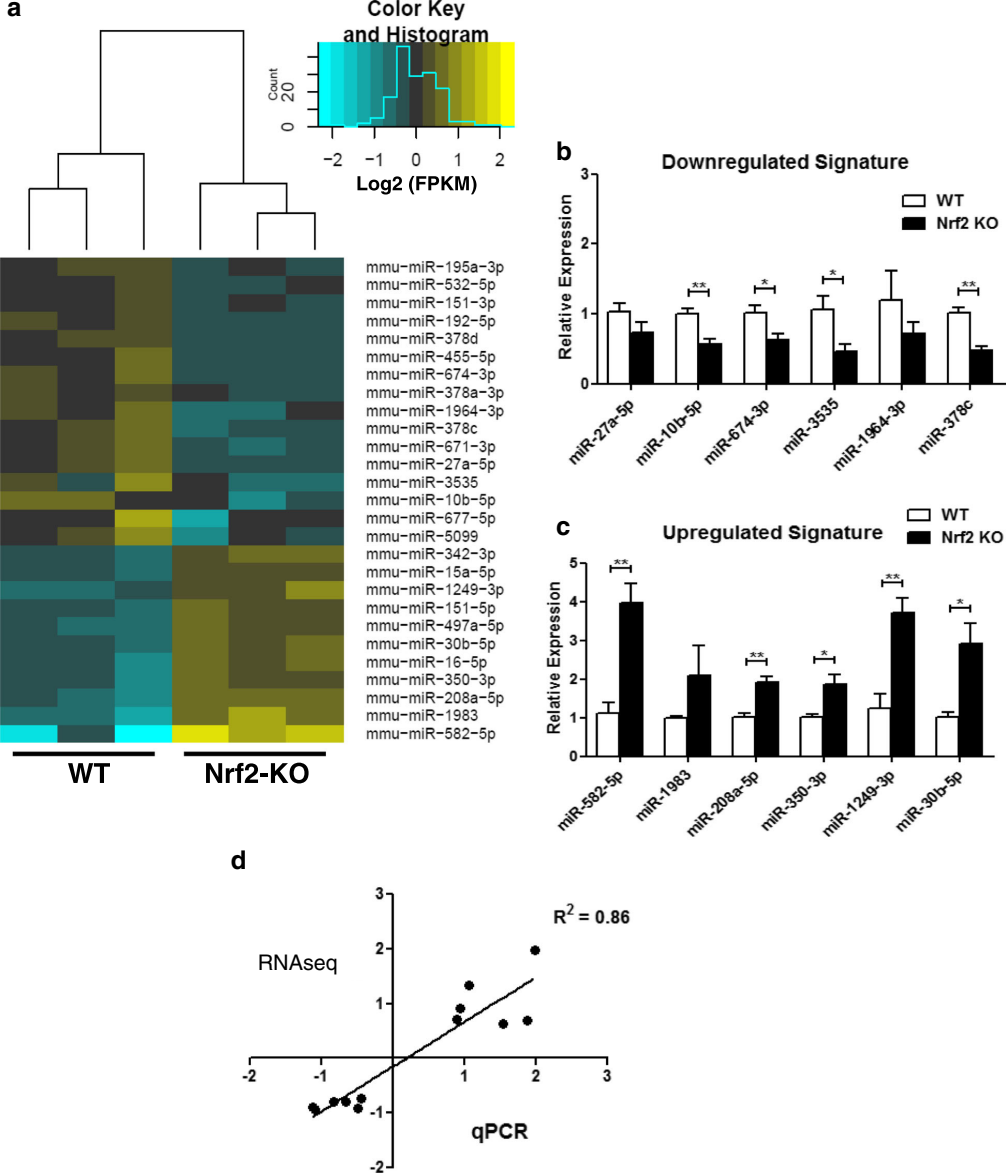
Myocardial microRNA Expression Profile: **a** Hierarchal clustering heat map showing all 27 differentially expressed microRNAs (miRNAs) in Nrf2 knockout hearts at baseline. **b-c** Real-time quantitative PCR validation (*n* = 4–6/group) of RNA sequencing (RNAseq) data using miRNA-specific primer assays for 6 upregulated and downregulated genes, respectively. **d** Log2 fold change (log2FC) derived from RNAseq correlated with mean log2FC obtained from qPCR experiments (*n* = 4–6/group). *p < 0.05, **p < 0.01, ***p < 0.001

The potential for cross-talk between miRNAs and Nrf2 signaling in the heart is unknown. Since stable miRNA expression is essential for myocardial development [45] and genetic manipulation of miRNAs produces cardiac pathology [46], we profiled basal miRNA expression patterns in Nrf2^−/−^ hearts using next generation RNAseq with small RNA sequencing libraries. Loss of Nrf2 in the heart resulted in 27 differentially expressed miRNAs. A total of 11 miRNAs exhibited increased expression while 16 were reduced in Nrf2^−/−^ hearts (Fig. 5a). Six robustly altered miRNAs from each of the upregulated and downregulated transcript pools were subsequently chosen for qPCR validation, which exhibited a strong correlation with the RNAseq dataset (Fig. 5d). Given the tissue specificity of miRNA responses [47], extrapolating biological significance of expression changes seen in Fig. 5b and c is challenging. Furthermore, many of the altered miRNAs observed in Nrf2^−/−^ hearts are not well characterized with respect to their specific role in cardiovascular pathology. However, the observation of reduced miR-10b-5p, miR-674-3p, miR-3535, and miR-378c expression upon Nrf2 ablation suggests that Nrf2 may be involved in the basal regulation of these miRNAs in the heart. In contrast to Nrf2 dependent miRNA downregulation (Fig. 5b), several miRNAs appeared to be induced upon Nrf2 knockout. Along these lines, we observed an approximate 2-fold (*p* < 0.01) increase in the expression of miR-208a-5p in the knockout myocardium (Fig. 5c). Encoded within the α-*MHC* gene, miR-208a directly regulates *Myh7b* and its intronic miRNA, miR-499 in the adult mouse heart. Upon stress, miR-208a dictates β*-MHC* transcription, thereby promoting hypertrophy and cardiogenic gene expression [48, 49]. However, β*-MHC*, *Myh7b* and *miR-499* expression were all unchanged in the Nrf2 deficient myocardium (data not presented). In addition to miR-208a, miR-350 has been shown to be an inducer of hypertrophy [50] and was found to be increased by nearly 2-fold (*p* < 0.05) in Nrf2^−/−^ hearts (Fig. 4c). However, the miR-350 transgenic mouse model used by Ge et al. exhibited a 9-fold increase in miR-350 expression levels [50]. Therefore, rather than serving as indices for pathology, miR-208a-5p and miR-350-3p expression increases in Nrf2^−/−^ mice may reflect susceptibility to adverse cardiac remodeling. In addition to these pro-hypertrophic miRNAs, miR-30b-5p and miR-582-5p expression was increased in the hearts of Nrf2 knockout mice (Fig. 5a). Importantly, miR-30b expression in cardiomyocytes contributes to survival and protection against oxidative stress induced mitochondrial fission [51], and miR-582-5p was recently shown to inhibit monocyte apoptosis [52]. Therefore, the 4-fold (*p* < 0.01) and 3-fold (*p* < 0.05) induction of miR-582-5p and miR-30b-5p (Fig. 5d) may also serve important homeostatic roles contributing to the maintenance of basal cardiac function in Nrf2^−/−^ mice. Altogether, the collection of miRNAs presented in Fig. 5 represents a novel list of candidates to examine in future studies investigating post-transcriptional regulation during myocardial stress.

### Potential miRNA:mRNA interactions at baseline

**Fig. 6:**
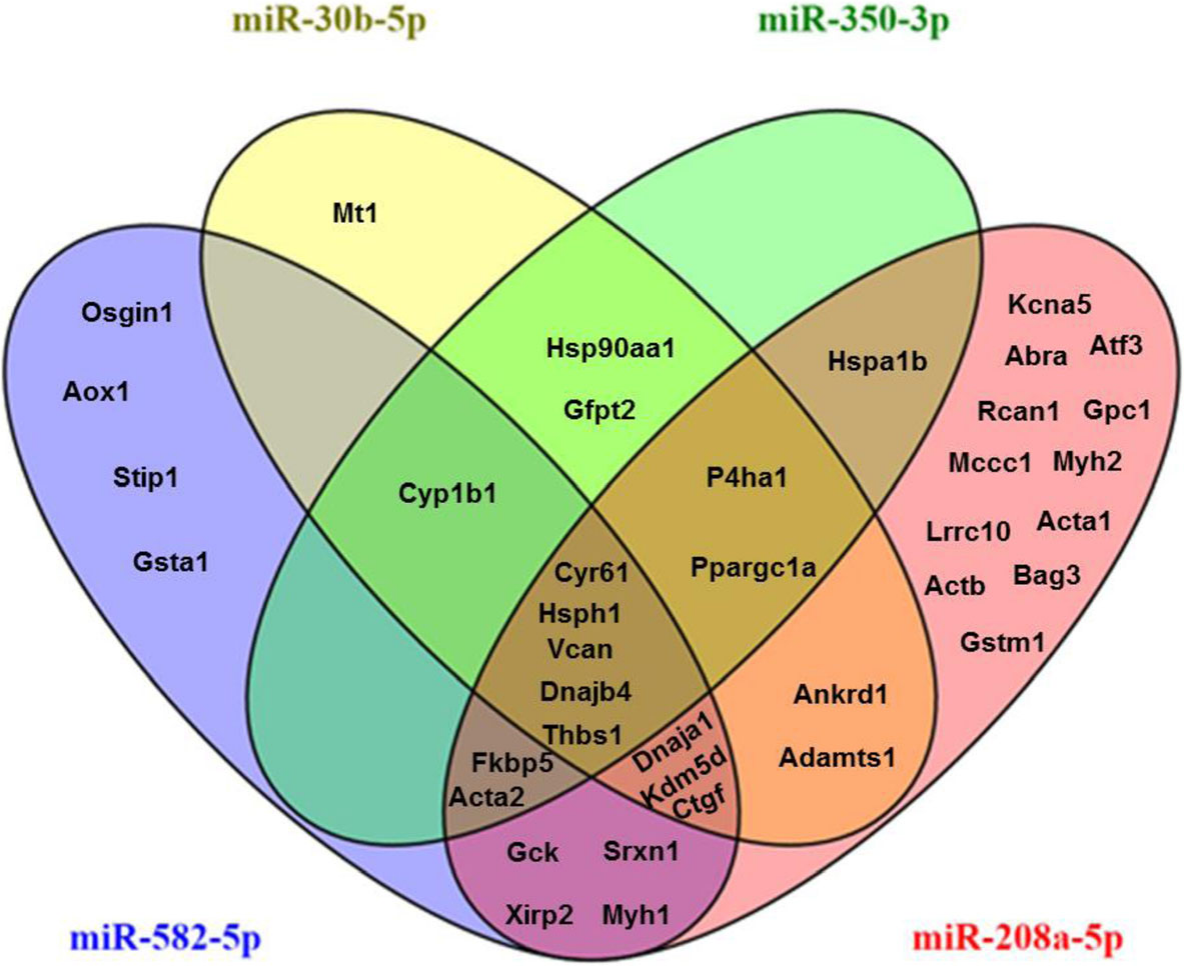
Potential miRNA:mRNA Interactions at Baseline: *In-silico* analysis of potential miRNA:mRNA interactions in the Nrf2 ablated myocardium. Given that the vast majority of mRNA expression changes occurred in the form of downregulation, bioinformatic predictions were made for upregulated miRNAs. To account for the varied computational aspects of commonly employed prediction tools, associations were made using the following 6 algorithms: miRANDA-mirSVR, TargetScan, MirTarget2 (miRDB), DIANA-microT-CDS, rna22, and PITA. A total of 39 downregulated DEGs contained potential miRNA recognition elements for the following upregulated miRNAs; miR-30b-5p, miR-350-3p, miR-582-5p, and miR-208a-5p. These miRNAs may synergistically regulate gene expression in knockout mice as 22 DEGs were shared by 2 or more miRNAs. 5 DEGs were shared by 3 upregulated miRNAs and an additional 5 DEGs harbored potential recognition elements for all 4 miRNAs

**Fig. 7:**
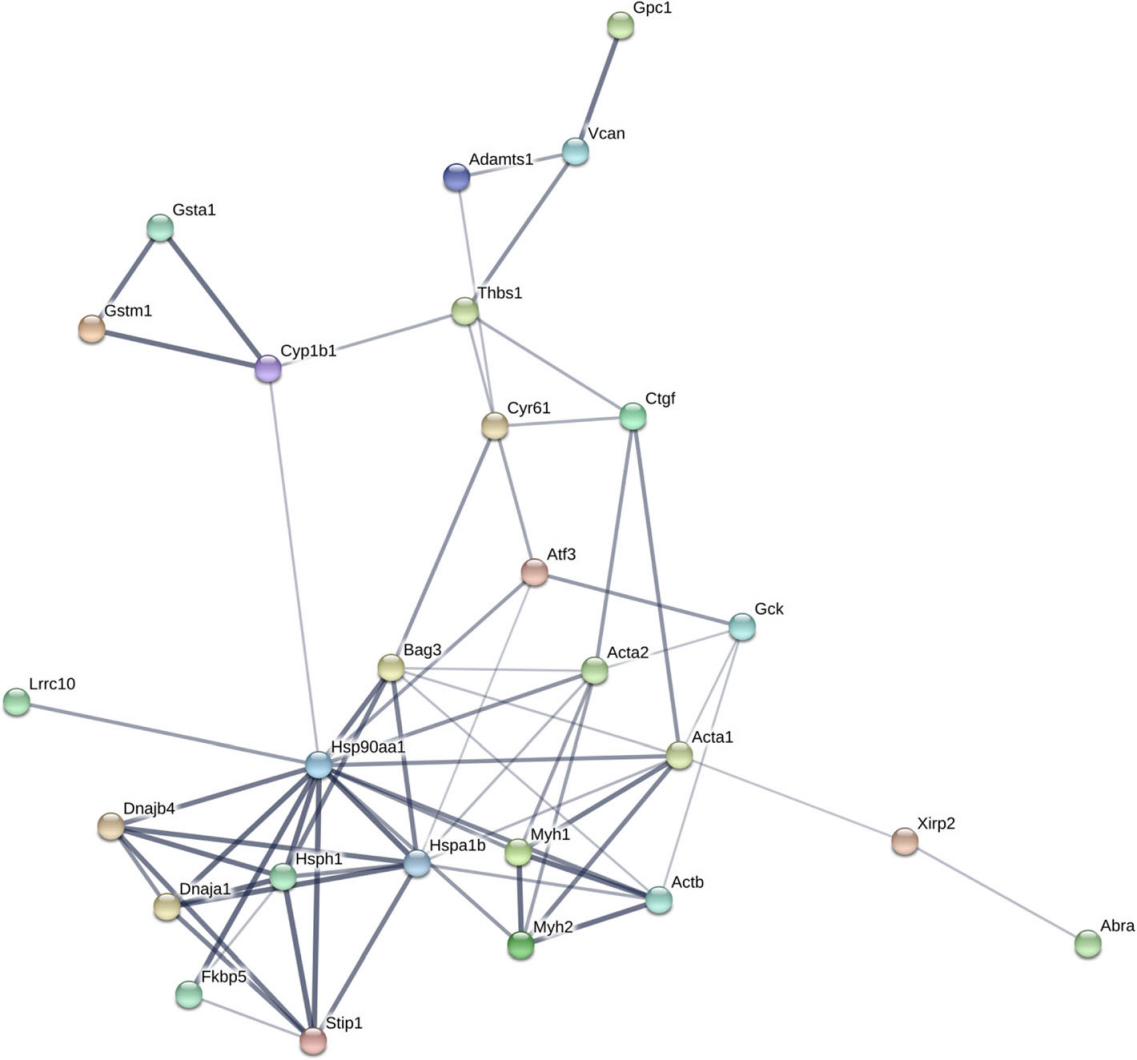
Molecular Interactions among Potential mRNA Targets: Gene network obtained from Search Tool for the Retrieval of Interacting Genes/Proteins (STRING) analysis. Genes implicated in miRNA:mRNA interactions at baseline were loaded in STRING using default interaction score settings. Disconnected nodes were omitted from the network display. Network edges denote the level of confidence with thicker lines denoting greater strength of data support

**Fig. 8:**
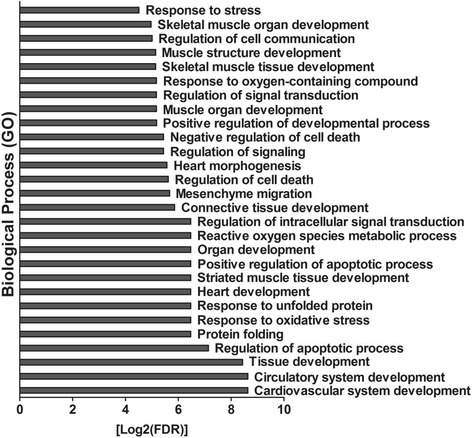
Biological Processes Implicated in miRNA:mRNA Interactome: Functional enrichment analysis of the STRING gene network revealing Gene Ontology (GO) based biological processes. All GO biological processes provided by STRING analysis are plotted according to their log2 transformed false discovery rate

To further understand the role of miRNA mediated post transcriptional regulation in the Nrf2 deficient myocardium, we integrated mRNA and miRNA sequencing data using miRNA target prediction algorithms. Since the majority of mRNA alterations occurred in the form of downregulation (Fig. 2b), we focused on relating this expression pattern with basally upregulated miRNAs in Nrf2^−/−^ mice. Due to the inherent limitations associated with bioinformatic prediction of miRNA targets [25], we meticulously searched for potential relationships using 6 algorithms; miRANDA-mirSVR, TargetScan, MirTarget2 (miRDB), DIANA-microT-CDS, rna22, and PITA. Using this approach, we discovered 39 transcripts potentially targeted by miR-30b-5p, miR-350-3p, miR-582-5p, and miR-208a-5p. Interestingly, bioinformatic target prediction revealed that 22 genes may potentially be regulated by two or more of these miRNAs. Further, 5 transcripts were commonly predicted to serve as targets for 3 miRNAs while an additional 5 mRNAs shared relationships with all 4 upregulated miRNAs (Fig. 6). Indeed, this may account for the substantial 2.8 and 4.9-fold decreases observed in *Ctgf* and *Thbs1*, respectively (Fig. 4c). In order to visualize molecular interactions among this gene network, we utilized Search Tool for the Retrieval of Interacting Genes/Proteins (STRING) software to understand important nodes which may be regulated by miRNAs (Fig. 7). Furthermore, STRING analysis allowed for the identification of enriched biological processes which may be influenced by these miRNA:mRNA interactions. Of note, the list of potential miRNA target genes downregulated in Nrf2^−/−^ hearts represented biological processes related to cardiovascular development, apoptosis regulation, protein folding, and oxidative stress (Fig. 8). These bioinformatic data provide initial insight into myocardial gene regulation by miRNAs in the Nrf2^−/−^ mouse, a previously unexplored area of research.

## Discussion

Nuclear factor erythroid 2 like 2 (NFE2L2/Nrf2) has received great attention as a cytoprotective transcription factor affording antioxidant defense through induction of antioxidant (ARE) and electrophile (EpRE) response elements. Although the Nrf2 homozygous null (Nrf2^−/−^) mouse is viable, it is highly susceptible to oxidative challenge [1]. Microarray based expression profiling of liver, intestine, and lung tissues have revealed compromised transcriptional responses in Nrf2^−/−^ mice [53–55]. However, the consequences of Nrf2 ablation on the myocardial transcriptome are unknown. Further, despite many examples of aberrant miRNA expression in cardiovascular diseases [22, 23], no reports exist on the impact or role of Nrf2 signaling over miRNA regulation in the heart [21]. Therefore, our comprehensive RNA sequencing efforts are the first attempt to study Nrf2-miRNA cross-talk in the heart. Our findings indicate that (i) loss of Nrf2 in the heart results in a modest downregulation of transcripts involved in mitochondrial function, redox homeostasis, metabolism, cardiac pathology, and protein folding, (ii) 27 miRNAs (11 up and 16 downregulated) are significantly altered and differentially regulated in the Nrf2 depleted myocardium, (iii) Nrf2 may either directly or indirectly regulate a sub-set of cardiac miRNAs, and (iv) miR-582-5p, miR-208a-5p, miR-350-3p and miR-30b-5p are likely to contribute to basally downregulated genes in Nrf2^−/−^ hearts. These high-throughput data suggest a relationship between several cardiac miRNAs and the Nrf2 pathway. Considering the sensitivity of Nrf2^−/−^ hearts to exogenous stressors [7, 8], these miRNAs could be novel candidates for future studies examining genetic regulation during myocardial stress and cardiac pathogenesis.

Loss of Nrf2 is commonly associated with reduced xenobiotic and antioxidant enzyme gene expression [56]. However, we have previously shown that the myocardium of young Nrf2^−/−^ mice retains basal antioxidant defense through steady expression of *Cat*, *Nqo1*, *Hmox1*/*HO-1*, *Gpx1*, *Gsr*, *Gclm*, and *Gclc* [7]. The results presented here expand on this notion and suggest that Nrf2 is dispensable to maintain basal antioxidant defense in an unstressed myocardium. The fact that so few ARE harboring genes were differentially expressed in Nrf2^−/−^ hearts implies that either these transcripts are basally quiescent, or regulated by other transcription factors. Indeed, both mechanisms appear evident upon examining fragments per kilobase of exon per million mapped reads (FPKM). While *Gclc* was profoundly low in both genotypes, *Cat* FPKM was sufficiently abundant in WT and Nrf2^−/−^ hearts (data not shown). In addition to preserved antioxidant transcript levels, reactive oxygen species (ROS) producing oxidative enzymes were surprisingly diminished in knockout hearts. Although the transcriptional mechanism responsible for the physiological status of antioxidants in Nrf2^−/−^ hearts is presently unknown, several reports have indicated that global loss of Nrf2 seems to have minimal effect on basal redox status of the cardiovascular system [7, 8]. Here, our transcriptomic sequencing indicates that Nrf2^−/−^ hearts possess enzymes necessary for glutathione biosynthesis and conjugation. Further, the marked increase in *Nnt* expression suggests active glutathione recycling at the mitochondria [33]. While our current and previous results suggest no signs of oxidative damage, a significant decrease was detected in 9 distinct gene members of the heat shock protein superfamily in Nrf2^−/−^ mice indicating an apparent stress-independent regulation of the basal heat shock network by Nrf2 in the heart. These findings support recent reports of overlapping function between Nrf2 and the canonical regulator of heat shock protein expression, Hsf1 [44].

Interestingly, Nrf2^−/−^ hearts displayed significant downregulation of sarcomeric genes. This is likely to be a result of compromised Gata4 and Hand2 cardiogenic transcriptional potential in knockout mice. The significant decrease in *Ankrd1* expression could negatively impact Gata4 transactivation in Nrf2^−/−^ mice as this is an important cofactor for Gata4 nuclear translocation [34]. Genes involved in fibrotic signaling were also robustly repressed in the Nrf2 ablated myocardium. Profoundly decreased *Thbs1* expression was observed in knockout hearts and this likely influenced latent TGF-β activation [57] as downstream targets *Ctgf* and *Acta2* [40–42] were markedly downregulated. Despite the striking decrease in pathogenic transcript abundance, limited developmental gene expression in Nrf2^−/−^ hearts may confer susceptibility upon exogenous stress. Indeed, we observed enriched biological processes indicative of developmental deficits in knockout mice following gene ontology analysis of differentially expressed mRNAs. We and others have documented that Nrf2 is essential to confer protection against pathological cardiac maladaptation via suppression of oxidant stress [58–61]. While ablation of both Nrf1 and Nrf2 result in embryonic lethality due to increase oxidative stress and apoptosis [62], loss of Nrf2 alone appears to be normal [62, 7]. However, mutations in Nrf2 SNPs leads to increase risk in congenital heart diseases [63, 64] and diabetic pregnancy showed increased cardiac malformations due to decreased Nrf2-antioxidant gene expressions [65]. Therefore, the cytoprotective role of Nrf2 in cardiac pathology may be associated with its ability to influence biological processes related to sarcomeric gene expression and myocardial development. To this end, the molecular consequences of diminished Gata4 and Hand2 expression in Nrf2^−/−^ hearts warrants further investigation.

As previously mentioned, cross-talk between miRNAs and the cardiac Nrf2 signaling cascade is unknown. Importantly, aberrant miRNA expression often underlies disease pathogenesis [19, 20] and ectopic miRNA activity in the heart can directly stimulate adverse remodeling [22, 23]. Our results suggest a post-transcriptional regulatory role for a sub-set of miRNAs in the Nrf2 ablated myocardium as 27 significantly altered genes were discovered. We believe these results can serve as a platform for future investigations into the role of miRNAs in myocardial redox state. Using next generation sequencing, we compared global miRNA abundance in WT and Nrf2^−/−^ hearts and discovered that knockout mice exhibited a downregulation in several miRNAs. These sequencing results were validated suggesting that Nrf2 may play a role in the basal regulation of myocardial miRNAs. Whether these miRNAs are directly or indirectly modulated by Nrf2 is currently not known. However, given the highly complex and multifactorial role of Nrf2 in redox homeostasis, metabolism, inflammation and xenobiotic defense, precisely defining these molecular relationships in the context of cardiac pathology warrants further investigation. While 16 miRNAs were repressed in Nrf2^−/−^ hearts, 11 additional miRNAs were upregulated as a result of Nrf2 deletion. Interestingly, Nrf2^−/−^ hearts displayed 2-fold induction of miR-208a-5p, a well-studied pro-hypertrophic miRNA [48, 49] and we believe this to be a compensatory increase for the downregulation of developmental genes in knockout hearts. To support this notion, a 3-fold increase in miR-208a is required for pathological cardiac remodeling [48]. Similarly, a 2-fold increase in miR-350-3p levels in Nrf2^−/−^ mice is unlikely to recapitulate the phenotype observed in miR-350 transgenic mice developing pathological hypertrophy [50]. In addition to these pro-hypertrophic miRNAs, miR-582-5p and miR-30b-5p levels were increased in the hearts of Nrf2^−/−^ mice. Recently, miR-582-5p was shown to inhibit monocyte apoptosis by targeting Foxo1 [52], a transcription factor that induces pro-apoptotic signaling in the heart conferring nitrosative and endoplasmic reticulum stress [66]. Similarly, miR-30b expression has been shown to play an essential role in survival and protection of cardiomyocytes against mitochondrial fission induced through hydrogen peroxide [51]. Therefore, the robust co-induction of miR-582-5p and miR-30b-5p in Nrf2^−/−^ mice could serve to maintain cardiomyocyte viability.

Upon integrating mRNA and miRNA expression data using bioinformatic target prediction [25], we discovered that upregulated miRNAs may substantially contribute to the downregulated mRNA transcriptome. Although certain limitations must be acknowledged when employing prediction algorithms, we were surprised at the number of overlapping transcripts shared by upregulated miRNAs. The fact that 22 DEGs contained potential recognition elements for 2 miRNAs, and 2 distinct subsets of 5 DEGs each matched with 3 and 4 upregulated miRNAs, respectively, suggests that miRNAs could play an important role in post-transcriptional regulation of genes in the Nrf2^−/−^ myocardium (Fig. 6). Importantly, these results lay the foundation for future efforts aimed at elucidating mechanisms whereby miRNAs influence myocardial redox homeostasis. Our future studies will expand on these findings to test how chronic oxidative stress marked by Nrf2 impairment alters cardiac miRNA abundance and maturation, thereby contributing to pathophysiology.

## Conclusions

Few studies have explored the role of cardiac miRNAs in myocardial redox regulation. Here, we have provided evidence for Nrf2 dependent regulation of a subset of cardiac miRNAs in the basal myocardium. Although experimental proof for direct mRNA targeting is outside the scope of this study, our results have laid the groundwork for future investigations into the molecular mechanisms of Nrf2 pathway sensitive miRNAs in the heart. Additionally, through unbiased RNA sequencing, we have provided a snapshot of the consequences of Nrf2 deletion on the cardiac mRNA transcriptome. These results provide extensive insight into genetic changes associated with Nrf2 loss of function and highlight novel miRNA candidates for future research on post-transcriptional regulation of genes governing cellular redox homeostasis*.* Limitations of the study include that it was impractical to validate each and every gene, an inherent problem with large-scale sequencing studies. Further, we have noted the potential for synergistic miRNA mediated regulation as several mRNA targets bear complementary sequences for more than one miRNA altered in Nrf2 knockout mice; however, the functional consequences of these regulatory effects at the protein level should be tested in future studies.

## Additional file

Additional file 1: Table S1. Complete List of Differentially Expressed Genes: All mRNAs determined to be significantly altered in Nrf2 knockout hearts following RNA sequencing analysis. Each gene symbol is presented with its official name, Log2 fold change (Log2FC) and adjusted *p*-value to account for false discovery rate (q-value). (DOCX 41 kb)

## Abbreviations

ARE/EpRE: Antioxidant/electrophile response element
DEGs: Differentially expressed genes
FC: Fold change
FDR: false discovery rate
miRNA: microRNA
mRNA: messenger RNA
Nrf2: Nuclear factor erythroid-derived 2-like 2
Nrf2^−/−^: Nrf2 knockout
qPCR: Quantitative PCR
RNAseq: RNA sequencing
ROS: Reactive oxygen species
STRING: Search Tool for the Retrieval of Interacting Genes/Proteins
