# Supplementary material for: Differential regulation of miRNA and mRNA expression in the myocardium of Nrf2 knockout mice
Source: BMC Genomics. 2017 Jul 3;18:509. doi: 10.1186/s12864-017-3875-3 (PMC5496330; doi:10.1186/s12864-017-3875-3)
Supplement: Additional file 1: Table S1. — Complete List of Differentially Expressed Genes: All mRNAs determined to be significantly altered in Nrf2 knockout hearts following RNA sequencing analysis. Each gene symbol is presented with its official name, Log2 fold change (Log2FC) and adjusted p-value to account for false discovery rate (q-value). (DOCX 41 kb) [file 12864_2017_3875_MOESM1_ESM.docx]

**Differential Regulation of miRNA and mRNA Expression in the Myocardium of Nrf2 Knockout Mice**

**Justin M. Quiles^1^, Madhusudhanan Narasimhan^2^, Gobinath Shanmugam^1^, Brett Milash^3^, John R. Hoidal^4^, Namakkal S. Rajasekaran^1,5,6#^.**

^1^Cardiac Aging & Redox Signaling Laboratory, Division of Molecular & Cellular Pathology, Department of Pathology, University of Alabama at Birmingham, Birmingham, AL-35294. ^2^Department of Pharmacology and Neuroscience, Texas Tech University Health Sciences Center, Lubbock, TX-79430. ^3^Huntsman Cancer Institute/^4^Division of Pulmonary/^5^Cardiovascular Medicine, Department of Medicine, University of Utah School of Medicine, Salt Lake City, UT-84132. ^5^Center for Free Radical Biology, University of Alabama at Birmingham, Birmingham, AL-35294.

**Running title**: miRNA vs. mRNA in Nrf2 knockout hearts

# Corresponding author

**Namakkal S. Rajasekaran, PhD.**

Cardiac Aging & Redox Signaling Laboratory

Center for Free Radical Biology, Division of Molecular & Cellular Pathology

Department of Pathology, UAB|University of Alabama at Birmingham

BMR2 Room 533|901 19th Street South|Birmingham, AL 35294-2180

Tel: 205.996.9839|Fax: 205.934.7447|Email: [rajnsr@uabmc.edu](mailto:rajnsr@uabmc.edu)

| **Complete List of Differentially Expressed Genes** | | | | | | |
| --- | --- | --- | --- | --- | --- | --- |
| **Gene Symbol** | | **Official Name** | **Log2FC** | | **q-Value** | |
| Xist | | inactive X specific transcripts | 2.17 | | 7.78E-184 | |
| Nfe2l2 | | nuclear factor, erythroid derived 2, like 2 | -3.17 | | 4.62E-178 | |
| Gm12913 | | predicted gene 12913 | -2.64 | | 3.59E-125 | |
| Thbs1 | | thrombospondin 1 | -1.81 | | 1.08E-64 | |
| Fmn1 | | formin 1 | -1.09 | | 5.70E-64 | |
| Aqp6 | | aquaporin 6 | -0.72 | | 7.94E-62 | |
| Gdpd3 | | glycerophosphodiester phosphodiesterase domain containing 3 | 1.07 | | 4.21E-55 | |
| Ctgf | | connective tissue growth factor | -1.43 | | 1.57E-45 | |
| Baiap2l1 | | BAI1-associated protein 2-like 1 | -1.08 | | 2.16E-45 | |
| Acta2 | | actin, alpha 2, smooth muscle, aorta | -1.05 | | 3.49E-40 | |
| AY036118 | | cDNA sequence AY036118 | 1.29 | | 3.94E-35 | |
| Mt2 | | metallothionein 2 | -1.06 | | 3.02E-27 | |
| mt-Tl1 | | mitochondrially encoded tRNA leucine 1 | -1.26 | | 4.18E-27 | |
| Cyr61 | | cysteine rich protein 61 | -1.02 | | 2.98E-22 | |
| Cd59a | | CD59a antigen | -1.05 | | 3.97E-21 | |
| Gm15564 | | predicted gene 15564 | 1.07 | | 5.71E-21 | |
| Gm13339 | | predicted gene 13339 | -1.07 | | 2.79E-20 | |
| Nnt | | nicotinamide nucleotide transhydrogenase | 1.04 | | 3.33E-20 | |
| Wif1 | | Wnt inhibitory factor 1 | -0.90 | | 4.40E-19 | |
| Hspa1b | | heat shock protein 1B | -1.12 | | 3.76E-17 | |
| Rrad | | Ras-related associated with diabetes | 0.95 | | 4.21E-17 | |
| Hspa1a | | heat shock protein 1A | -1.07 | | 7.88E-17 | |
| Hbegf | | heparin-binding EGF-like growth factor | -0.64 | | 5.72E-16 | |
| Lrtm1 | | leucine-rich repeats and transmembrane domains 1 | -0.96 | | 8.25E-16 | |
| Cpxm2 | | carboxypeptidase X 2 (M14 family) | -1.17 | | 1.12E-15 | |
| Cdh4 | | cadherin 4 | -0.92 | | 2.85E-15 | |
| Atf3 | | activating transcription factor 3 | -0.91 | | 2.85E-15 | |
| Rcan1 | | regulator of calcineurin 1 | -0.81 | | 2.85E-15 | |
| Ppm1k | | protein phosphatase 1K (PP2C domain containing) | 0.80 | | 4.42E-15 | |
| Cyp1b1 | | cytochrome P450, family 1, subfamily b, polypeptide 1 | -0.86 | | 5.19E-15 | |
| Aox1 | | aldehyde oxidase 1 | -0.88 | | 1.43E-14 | |
| Pyurf | | Pigy upstream reading frame | -0.83 | | 2.20E-14 | |
| Cdkn1a | | cyclin-dependent kinase inhibitor 1A (P21) | -0.95 | | 8.23E-14 | |
| Mccc1 | | methylcrotonoyl-Coenzyme A carboxylase 1 (alpha) | -0.88 | | 1.09E-13 | |
| Osbpl6 | | oxysterol binding protein-like 6 | -0.82 | | 1.20E-13 | |
| Gstm1 | | glutathione S-transferase, mu 1 | -0.88 | | 1.65E-13 | |
| Xirp2 | | xin actin-binding repeat containing 2 | -0.86 | | 7.33E-13 | |
| Bcl6 | | B cell leukemia/lymphoma 6 | -0.80 | | 9.24E-13 | |
| **Complete List of Differentially Expressed Genes (continued)** | | | | | | |
| **Gene Symbol** | **Official Name** | | | **Log2FC** | | **q-Value** |
| Gm4707 | predicted gene 4707 | | | 0.81 | | 1.33E-12 |
| Gck | glucokinase | | | -0.88 | | 1.42E-12 |
| mt-Tm | mitochondrially encoded tRNA methionine | | | -0.86 | | 2.18E-12 |
| March3 | membrane-associated ring finger (C3HC4) 3 | | | -0.59 | | 2.30E-12 |
| Hand2 | heart and neural crest derivatives expressed transcript 2 | | | -0.87 | | 3.35E-12 |
| Slc17a7 | solute carrier family 17 (sodium-dependent inorganic phosphate cotransporter), member 7 | | | -0.76 | | 7.52E-12 |
| Mt1 | metallothionein 1 | | | -0.88 | | 8.29E-12 |
| mt-Rnr2 | mitochondrially encoded 16S rRNA | | | -0.90 | | 1.45E-11 |
| mt-Te | mitochondrially encoded tRNA glutamic acid | | | -0.82 | | 2.25E-11 |
| \| Xirp1 \| \| xin actin-binding repeat containing 1 \| -0.67 \| \| 3.21E-11 \| \| \| --- \| --- \| --- \| --- \| --- \| --- \| --- \| \| Myh2 \| \| myosin, heavy polypeptide 2, skeletal muscle, adult \| -0.62 \| \| 3.21E-11 \| \| \| Ccrn4l \| \| CCR4 carbon catabolite repression 4-like (S. cerevisiae) \| -1.09 \| \| 4.38E-11 \| \| \| Ptgds \| \| prostaglandin D2 synthase (brain) \| -0.94 \| \| 5.67E-11 \| \| \| Myc \| \| myelocytomatosis oncogene \| -0.73 \| \| 1.20E-10 \| \| \| mt-Nd6 \| \| mitochondrially encoded NADH dehydrogenase 6 \| -0.79 \| \| 1.73E-10 \| \| \| Gsta1 \| \| glutathione S-transferase, alpha 1 (Ya) \| -0.88 \| \| 2.64E-10 \| \| \| mt-Tq \| \| mitochondrially encoded tRNA glutamine \| -0.82 \| \| 3.40E-10 \| \| \| Ankrd23 \| \| ankyrin repeat domain 23 \| -0.75 \| \| 3.52E-10 \| \| \| Aplnr \| \| apelin receptor \| 0.61 \| \| 5.30E-10 \| \| \| Mgst1 \| \| microsomal glutathione S-transferase 1 \| -0.80 \| \| 5.47E-10 \| \| \| H2-Q6 \| \| histocompatibility 2, Q region locus 6 \| 0.78 \| \| 8.68E-10 \| \| \| Icam1 \| \| intercellular adhesion molecule 1 \| -0.71 \| \| 9.90E-10 \| \| \| Hsph1 \| \| heat shock 105kDa/110kDa protein 1 \| -0.93 \| \| 1.04E-09 \| \| \| Gm21685 \| \| predicted gene, 21685 \| -0.73 \| \| 1.06E-09 \| \| \| Lrrc10 \| \| leucine rich repeat containing 10 \| -0.61 \| \| 1.06E-09 \| \| \| Cebpd \| \| CCAAT/enhancer binding protein (C/EBP), delta \| -0.66 \| \| 1.36E-09 \| \| \| 2810474O19Rik \| \| RIKEN cDNA 2810474O19 gene \| -0.92 \| \| 1.84E-09 \| \| \| Acta1 \| \| actin, alpha 1, skeletal muscle \| -0.70 \| \| 2.21E-09 \| \| \| Gm10222 \| \| predicted gene 10222 \| -0.72 \| \| 2.56E-09 \| \| \| Akap5 \| \| A kinase (PRKA) anchor protein 5 \| 0.64 \| \| 3.28E-09 \| \| \| Ankrd1 \| \| ankyrin repeat domain 1 (cardiac muscle) \| -0.63 \| \| 3.38E-09 \| \| \| mt-Nd4l \| \| mitochondrially encoded NADH dehydrogenase 4L \| -0.71 \| \| 3.88E-09 \| \| \| Osgin1 \| \| oxidative stress induced growth inhibitor 1 \| -0.64 \| \| 4.26E-09 \| \| \| Tmem144 \| \| transmembrane protein 144 \| -0.81 \| \| 4.41E-09 \| \| \| Eif2s3y \| \| eukaryotic translation initiation factor 2, subunit 3, structural gene Y-linked \| -1.63 \| \| 4.41E-09 \| \| \| Ccl9 \| \| chemokine (C-C motif) ligand 9 \| -0.67 \| \| 5.52E-09 \| \| \| Serpine1 \| \| serine (or cysteine) peptidase inhibitor, clade E, member 1 \| -0.69 \| \| 5.58E-09 \| \| \| mt-Rnr1 \| \| mitochondrially encoded 12S rRNA \| -0.77 \| \| 5.99E-09 \| \| \| **Complete List of Differentially Expressed Genes (continued)** \| \| \| \| \| \| \| \| **Gene Symbol** \| \| **Official Name** \| **Log2FC** \| \| **q-Value** \| \| \| Ifi205 \| \| interferon activated gene 205 \| -0.69 \| \| 6.32E-09 \| \| \| Vcan \| \| versican \| -0.66 \| \| 7.31E-09 \| \| \| Atp5e \| \| ATP synthase, H+ transporting, mitochondrial F1 complex, epsilon subunit \| 0.71 \| \| 7.47E-09 \| \| \| Osmr \| \| oncostatin M receptor \| -0.70 \| \| 9.61E-09 \| \| \| Gm15920 \| \| predicted gene 15920 \| -0.70 \| \| 1.05E-08 \| \| \| Il15 \| \| interleukin 15 \| 0.84 \| \| 1.57E-08 \| \| \| Plbd1 \| \| phospholipase B domain containing 1 \| -0.76 \| \| 1.62E-08 \| \| \| Adamts1 \| \| a disintegrin-like and metallopeptidase (reprolysin type) with thrombospondin type 1 motif, 1 \| -0.63 \| \| 2.00E-08 \| \| \| Gm11408 \| \| predicted gene 11408 \| -0.70 \| \| 2.11E-08 \| \| \| Taf5 \| \| TAF5 RNA polymerase II, TATA box binding protein (TBP)-associated factor \| -0.71 \| \| 2.45E-08 \| \| \| Sort1 \| \| sortilin 1 \| -0.66 \| \| 2.96E-08 \| \| \| Rab6b \| \| RAB6B, member RAS oncogene family \| 0.99 \| \| 3.07E-08 \| \| \| St3gal5 \| \| ST3 beta-galactoside alpha-2,3-sialyltransferase 5 \| -0.71 \| \| 3.41E-08 \| \| \| Rftn2 \| \| raftlin family member 2 \| 0.69 \| \| 5.99E-08 \| \| \| Gfpt2 \| \| glutamine fructose-6-phosphate transaminase 2 \| -0.70 \| \| 7.06E-08 \| \| \| Srxn1 \| \| sulfiredoxin 1 homolog (S. cerevisiae) \| -0.65 \| \| 7.49E-08 \| \| \| D3Ertd751e \| \| DNA segment, Chr 3, ERATO Doi 751, expressed \| -0.65 \| \| 1.21E-07 \| \| \| Stip1 \| \| stress-induced phosphoprotein 1 \| -0.74 \| \| 1.24E-07 \| \| \| Nr4a3 \| \| nuclear receptor subfamily 4, group A, member 3 \| \| -1.17 \| 1.74E-07 \| \| \| \| Il4ra \| \| interleukin 4 receptor, alpha \| \| -0.61 \| 2.49E-07 \| \| \| \| Fam107a \| \| family with sequence similarity 107, member A \| \| -0.82 \| 2.92E-07 \| \| \| \| Ccl2 \| \| chemokine (C-C motif) ligand 2 \| \| -0.61 \| 3.23E-07 \| \| \| \| Actb \| \| actin, beta \| \| -0.66 \| 4.04E-07 \| \| \| \| Fkbp5 \| \| FK506 binding protein 5 \| \| -0.75 \| 4.16E-07 \| \| \| \| Sik1 \| \| salt inducible kinase 1 \| \| -0.64 \| 4.65E-07 \| \| \| \| Nudt6 \| \| nudix (nucleoside diphosphate linked moiety X)-type motif 6 \| \| -0.61 \| 6.59E-07 \| \| \| \| Ifi204 \| \| interferon activated gene 204 \| \| -0.63 \| 6.79E-07 \| \| \| \| Mapt \| \| microtubule-associated protein tau \| \| -0.63 \| 7.34E-07 \| \| \| \| Gm13394 \| \| predicted gene 13394 \| \| -0.58 \| 8.50E-07 \| \| \| \| Rps21 \| \| ribosomal protein S21 \| \| 0.65 \| 9.98E-07 \| \| \| \| Myh1 \| \| myosin, heavy polypeptide 1, skeletal muscle, adult \| \| -0.58 \| 9.98E-07 \| \| \| \| Gpc1 \| \| glypican 1 \| \| -0.62 \| 1.04E-06 \| \| \| \| Serpina3n \| \| serine (or cysteine) peptidase inhibitor, clade A, member 3N \| \| -0.63 \| 1.10E-06 \| \| \| \| Frmd5 \| \| FERM domain containing 5 \| \| -0.91 \| 1.24E-06 \| \| \| \| Ddx3y \| \| DEAD (Asp-Glu-Ala-Asp) box polypeptide 3, Y-linked \| \| -1.86 \| 1.35E-06 \| \| \| \| Creb3l1 \| \| cAMP responsive element binding protein 3-like 1 \| \| -0.61 \| 1.54E-06 \| \| \| \| H2-Q4 \| \| histocompatibility 2, Q region locus 4 \| \| 0.60 \| 1.73E-06 \| \| \| \| Clec4d \| \| C-type lectin domain family 4, member d \| \| -0.58 \| 1.73E-06 \| \| \| \| **Complete List of Differentially Expressed Genes (continued)** \| \| \| \| \| \| \| \| \| **Gene Symbol** \| \| **Official Name** \| \| **Log2FC** \| **q-Value** \| \| \| \| H2-K1 \| \| histocompatibility 2, K1, K region \| \| 0.59 \| 2.33E-06 \| \| \| \| Bola3 \| \| bolA-like 3 (E. coli) \| \| -0.61 \| 2.40E-06 \| \| \| \| mt-Nd3 \| \| mitochondrially encoded NADH dehydrogenase 3 \| \| -0.62 \| 2.49E-06 \| \| \| \| Gm6977 \| \| predicted gene 6977 \| \| -0.58 \| 2.54E-06 \| \| \| \| Zfp36 \| \| zinc finger protein 36 \| \| -0.58 \| 2.58E-06 \| \| \| \| Dbh \| \| dopamine beta hydroxylase \| \| -0.59 \| 4.18E-06 \| \| \| \| Gm13341 \| \| predicted gene 13341 \| \| -0.58 \| 5.54E-06 \| \| \| \| Litaf \| \| LPS-induced TN factor \| \| -0.59 \| 6.15E-06 \| \| \| \| Smim5 \| \| small integral membrane protein 5 \| \| 0.59 \| 6.41E-06 \| \| \| \| Cmklr1 \| \| chemokine-like receptor 1 \| \| -0.65 \| 6.55E-06 \| \| \| \| Hspb1 \| \| heat shock protein 1 \| \| -0.75 \| 8.56E-06 \| \| \| \| Abra \| \| actin-binding Rho activating protein \| \| -0.77 \| 1.05E-05 \| \| \| \| Cd200 \| \| CD200 antigen \| \| 0.62 \| 1.17E-05 \| \| \| \| S1pr1 \| \| sphingosine-1-phosphate receptor 1 \| \| -0.69 \| 1.19E-05 \| \| \| \| Hsp90aa1 \| \| heat shock protein 90, alpha (cytosolic), class A member 1 \| \| -0.81 \| 1.79E-05 \| \| \| \| F830016B08Rik \| \| RIKEN cDNA F830016B08 gene \| \| 0.60 \| 1.80E-05 \| \| \| \| Ell2 \| \| elongation factor RNA polymerase II 2 \| \| -0.64 \| 1.80E-05 \| \| \| \| Gm5844 \| \| predicted gene 5844 \| \| -0.76 \| 3.70E-05 \| \| \| \| Lgr6 \| \| leucine-rich repeat-containing G protein-coupled receptor 6 \| \| 0.72 \| 4.36E-05 \| \| \| \| Gm12346 \| \| predicted gene 12346 \| \| -0.76 \| 5.63E-05 \| \| \| \| Bag3 \| \| BCL2-associated athanogene 3 \| \| -0.61 \| 8.25E-05 \| \| \| \| Cebpb \| \| CCAAT/enhancer binding protein (C/EBP), beta \| \| -0.63 \| 1.28E-04 \| \| \| \| Olfml2b \| \| olfactomedin-like 2B \| \| -0.60 \| 2.88E-04 \| \| \| \| Gm23935 \| \| predicted gene, 23935 \| \| 0.58 \| 3.23E-04 \| \| \| \| Cpeb4 \| \| cytoplasmic polyadenylation element binding protein 4 \| \| -0.62 \| 3.51E-04 \| \| \| \| Kcna5 \| \| potassium voltage-gated channel, shaker-related subfamily, member 5 \| \| -0.61 \| 4.73E-04 \| \| \| \| Map3k6 \| \| mitogen-activated protein kinase kinase kinase 6 \| \| -0.60 \| \| \| 6.95E-04 \| \| P4ha1 \| \| procollagen-proline, 2-oxoglutarate 4-dioxygenase (proline 4-hydroxylase), alpha 1 polypeptide \| \| -0.67 \| 8.61E-04 \| \| \| \| Uty \| \| ubiquitously transcribed tetratricopeptide repeat gene, Y chromosome \| \| -1.31 \| 1.07E-03 \| \| \| \| 8430408G22Rik \| \| RIKEN cDNA 8430408G22 gene \| \| -0.73 \| 1.59E-03 \| \| \| \| Chordc1 \| \| cysteine and histidine-rich domain (CHORD)-containing, zinc-binding protein 1 \| \| -0.58 \| 1.79E-03 \| \| \| \| Dnajb4 \| \| DnaJ (Hsp40) homolog, subfamily B, member 4 \| \| -0.60 \| 3.46E-03 \| \| \| \| Ppargc1a \| \| peroxisome proliferative activated receptor, gamma, coactivator 1 alpha \| \| -0.83 \| 4.32E-03 \| \| \| \| Hspa8 \| \| heat shock protein 8 \| \| -0.62 \| 1.47E-02 \| \| \| \| Kdm5d \| \| lysine (K)-specific demethylase 5D \| \| -0.88 \| 2.51E-02 \| \| \| \| Dnaja1 \| \| DnaJ (Hsp40) homolog, subfamily A, member 1 \| \| -0.63 \| 2.91E-02 \| \| \| | | | | | | |
